# Biotests and Biosensors for Ecotoxicology of Metal Oxide Nanoparticles: A Minireview

**DOI:** 10.3390/s8085153

**Published:** 2008-08-28

**Authors:** Anne Kahru, Henri-Charles Dubourguier, Irina Blinova, Angela Ivask, Kaja Kasemets

**Affiliations:** 1 Laboratory of Molecular Genetics, National Institute of Chemical Physics and Biophysics, Akadeemia tee 23, Tallinn 12618, Estonia; 2 Estonian University of Life Sciences, Kreutzwaldi 5, Tartu 51014, Estonia

**Keywords:** ZnO, CuO, TiO_2_, Aquatic toxicity, Bioavailability, Recombinant sensor bacteria, 3Rs, Daphnia magna, Thamnocephalus platyurus, Pseudokirchneriella subcapitata (Selenastrum capricornutum), Tetrahymena thermophila, Vibrio fischeri

## Abstract

Nanotechnologies have become a significant priority worldwide. Several manufactured nanoparticles - particles with one dimension less than 100 nm - are increasingly used in consumer products. At nanosize range, the properties of materials differ substantially from bulk materials of the same composition, mostly due to the increased specific surface area and reactivity, which may lead to increased bioavailability and toxicity. Thus, for the assessment of sustainability of nanotechnologies, hazards of manufactured nanoparticles have to be studied. Despite all the above mentioned, the data on the potential environmental effects of nanoparticles are rare. This mini-review is summarizing the emerging information on different aspects of ecotoxicological hazard of metal oxide nanoparticles, focusing on TiO_2_, ZnO and CuO. Various biotests that have been successfully used for evaluation of ecotoxic properties of pollutants to invertebrates, algae and bacteria and now increasingly applied for evaluation of hazard of nanoparticles at different levels of the aquatic food-web are discussed. Knowing the benefits and potential drawbacks of these systems, a suite of tests for evaluation of environmental hazard of nanoparticles is proposed. Special attention is paid to the influence of particle solubility and to recombinant metal-sensing bacteria as powerful tools for quantification of metal bioavailability. Using recombinant metal-specific bacterial biosensors and multitrophic ecotoxicity assays in tandem will create new scientific knowledge on the respective role of ionic species and of particles in toxicity of metal oxide nanoparticles.

## 1. Introduction

It is remarkable that although environmental research and protection efforts as well as health-related investments are constantly increasing, the current world witnesses the significant increase of neurodegenerative and cardiovascular diseases, allergies, cancers and also a clear trend towards drastic deterioration of natural ecosystems. That was the main reason of the introduction of the E.U.'s new REACH (Registration, Evaluation and Authorizations of Chemicals) chemical policy [1]. Presently, the current chemical regulations (including REACH) fail to address the environmental, health, and safety risks posed by nanomaterials/particles but given the urgent need for the evaluation of their biological effects, this is actively debated, also at the E.U. level [2]. Nanotechnologies have become a significant priority in many countries. Nanoparticles are defined as natural or manufactured particles with one dimension less than 100 nm. Some natural particles are of nano-scale such as colloidal humus [3, 4] and ultrafine particles in atmospheric emissions [5]. Environmental nanoparticles are commonly formed as either weathering byproducts of minerals, as biogenic products of microbial activity, or as growth nuclei in super-saturated fluids [6]. Manufactured nanoparticles can be inorganic like nanopowders of metal oxides and metal salts like CdS (quantum dots) or organic chemicals and polymers like dendrimers [7].

According to “The Nanotechnology Consumer Products Inventory” [8] the most common material mentioned in the product descriptions was carbon (29 products) which included fullerenes and nanotubes. Silver was the second most referenced (25 products), followed by silica (14), titanium dioxide (8), zinc oxide (8), and cerium oxide (1). Among potential environmental applications of nanoparticles, remediation of contaminated groundwater with nanoscale iron is one of the most prominent examples [9, 10]. Regarding personal-care products, nanoparticles of titanium dioxide and zinc oxide are included in toothpaste, beauty products, sunscreens [11] and textiles [12]. Metal oxide-based nanomaterials and/or nanoparticles are also increasingly used in fillers, opacifiers, ceramics, coatings, catalysts, semiconductors, microelectronics, prosthetic implants and drug carriers [7, 13]. Photocatalytic properties of TiO_2_ may be used for solar-driven self-cleaning coatings [14] and for biocidal/antiproliferative applications [15]. Copper oxide nanoparticles have potential to replace noble metal catalysts for carbon monoxide oxidation [16] and CuO nanoparticle suspension (nanofluid) has excellent thermal conductivity for it to be used as a heat transfer fluid in machine tools [17].

In the form of manufacturing and household waste the metal oxide nanoparticles are likely to end up in natural water bodies. For perspective on potential “nanopollution”, one may consider that 2 g of 100 nm size nanoparticles contains enough material to provide every human worldwide with 300,000 particles each [18]. Decrease in particle size changes the physicochemical and structural properties of particles and in the case of nanoparticles that is responsible for increased bioavailability and toxic effects [7]. Nanoparticles can cross even the strongest biological barriers such as blood-brain barrier [19, 20]. As an example, Oberdörster *et al.* [19] showed that exposure to fullerenes (C_60_) caused oxidative damage in the brain of fish. Despite that, nanosized materials were till recently treated as variations of the technical material or existing formulation and thus not requiring separate registration [21].

Due to the current commercial development of nanotechnology, the occupational and public exposure to nanoparticles is supposed to increase dramatically in the coming years as well as their potential release in the environment. Thus, the studies on safety and (eco)toxicity of nanoparticles are of extreme importance in order to support the sustainable development of nanotechnology.

**Figure 1.**
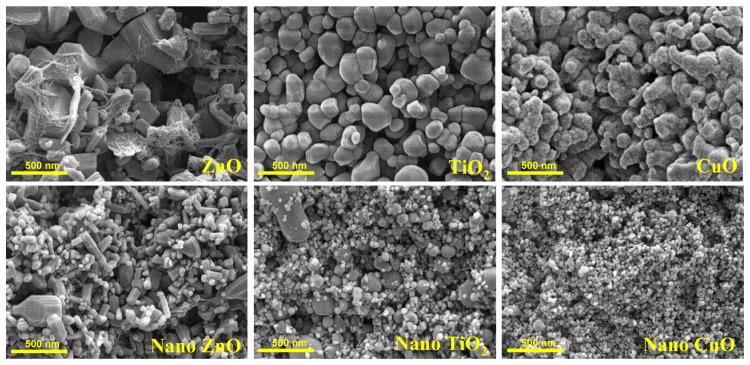
Scanning electron microscopy of ZnO, TiO_2_ and CuO particles. The bulk form of TiO_2_ was purchased from Riedel-de Haen, ZnO from Fluka and CuO from Alfa Aesar. Nanosized metal oxides were purchased from Sigma–Aldrich with advertised particles sizes of 25-70 nm for nano TiO_2_, 50-70 nm for nano ZnO and mean ∼30 nm for nano CuO. Observations were made using Zeiss Digital Scanning Electron Microscope (DSM 982 Gemini).

Despite of the rapid increase of nanotoxicological peer-reviewed papers published, most of the data has been obtained on limited types of particles and mostly on *in vitro* cell cultures or *in vivo* respiratory exposures on rodents [22]. The knowledge on their potential harmful effects on the environment remains poorly documented. There are few data available on the effects of engineered nanoparticles on algae, plants, and fungi [23] as well as on aquatic invertebrates. Overall there are currently less than 50 open peer-reviewed ecotoxicity studies on environmentally relevant species [24]. Nanomaterial-wise, the existing ecotoxicological information mainly concerns toxicity data on fullerenes, carbon nanotubes and TiO_2_ [19, 24-26]. As an indicator concerning various metal oxide nanoparticles, a bibliometric search on 4th July 2008 in ISI Web of Science showed that although there were more than 150 hits in combining keywords on respective nano metal oxides and “toxic*” but only 10 hits for “ecotoxic” (Table 1). Thus, the current minireview summarizes existing literature on ecotoxic effects of ZnO, TiO_2_ and CuO nanoparticles (Figure 1), especially to aquatic invertebrates, algae and bacteria.

## 2. Toxicity Mechanisms of Metal Oxide Nanoparticles

### 2.1. Reactive Oxygen Species (ROS)

Currently, the best-developed paradigm for nanoparticles toxicity for eukaryotes is generation of reactive oxygen species (ROS) [7, 27]. ROS production is especially relevant in the case of nanoparticles with photocatalytic properties such as TiO_2_ [28]. ROS have been shown to damage cellular lipids, carbohydrates, proteins and DNA [29] and leading to inflammation and oxidative stress response [27, 30, 31]. Lipid peroxidation is considered the most dangerous since it leads to alterations in cell membrane properties which in its turn disrupts vital cellular functions [21, 32, 33]. The oxidative stress mechanisms are also linked to a number of human pathologies and aging [34, 35]. Experimentally, nanoparticles have been shown to induce oxidative stress responses *in vitro* in keratinocytes, macrophages and blood monocytes [36, 37]. Recent *in vitro* data also revealed ROS-mediated potential neurotoxicity of nano TiO_2_ [38]. In human lung epithelial cells, the chemical composition of nanoparticles was the most decisive factor determining the formation of ROS in exposed cells to iron-, cobalt-, manganese-, and titanium-containing silica nanoparticles and respective pure metal oxide nanoparticles [39]. During their life cycle, engineered nanoparticles might also produce ROS upon interactions with abiotic and biotic environmental factors. Indeed, damaging effects of TiO_2_ nanoparticles on bacteria have been shown to be enhanced by sunlight or UV illumination [40]. TiO_2_ nanoparticles in combination with UV-light have been shown to inactivate algae *Anabaena*, *Microcystis* and *Melosira* [41] and have been shown to destroy the cell surface architecture of blue-green algae *Chroococcus* sp. [42]. However, TiO_2_ nanoparticles have shown toxicity to *Bacillus subtilis* and *Escherichia coli* also in the dark [40]. Analogously, in fish cells *in vitro* hydroxyl radicals were generated by TiO_2_ nanoparticles also in the absence of ultraviolet light [43]. ROS could be also involved in toxicity of ZnO nanoparticles to bacteria as recently shown for *Escherichia coli* [44]. Heavy metals induce oxidative stress in algae via different types of ROS-generating mechanisms [45] but the role of ROS in toxicity of (metal-containing) nanoparticles in algae remains largely unknown.

### 2.2. Release of Metal Ions

In the case of metal-containing nanoparticles, also the release of metal ions and their speciation may be a key factor in their (eco)toxicity. Indeed, as shown for oxide nanoparticles (incl. TiO_2_ and ZnO) using *in vitro* cell cultures, solubility of those nanoparticles strongly influenced their cytotoxicity [46]. In human lung epithelial cells *in vitro*, Limbach *et al.* [39] have recently shown that partially soluble nanoparticles such as cobalt oxide and manganese oxide may be taken up into cells by a Trojan-horse type mechanism, i.e. metal oxide nanoparticles entered the cells but not the respective ionic forms. The induced oxidative stress was therefore remarkably higher than in the case of ions of the corresponding metals for which the transport is controlled. Indeed, the metal oxide nanoparticles once in the cell may dissolve releasing higher damaging concentrations of metal ions within the cell. Liberation of cytotoxic amounts of Cd in physiological conditions has also been shown for CdSe quantum dots [47]. Therefore, it has been stressed that water solubility of nanoparticles has to be incorporated into the environmental risk assessment models of nanoparticles in addition to other key physico-chemical characteristics relevant to nanoparticles [48].

Bacteria have no internalization mechanisms for supramolecular and colloidal particles. However, <5 nm CdSe and CdSe/ZnS quantum dots have been shown to enter the bacterial cells [49], but not ellipsoidal shaped carboxylated and biotinylated CdSe/ZnS QD with minor and major axis of 12 nm and 6 nm, respectively [50]. Differently from bacteria, protists and metazoans have highly developed systems for internalization of nano and microscale particles. In addition, due to their large surface area, nanoparticles have been shown to sorb heavy metals, PAHs, quinolines [51, 52]. Baun *et al.* [53] showed that the toxicity of phenanthrene for *Daphnia magna* was increased by 60% in the presence of C60 aggregates and that sorbed phenanthrene was available for the organisms. Thus nanoparticles not only act as transfer vectors in the environment, but they also facilitate the entry of nanoparticle-sorbed pollutants into cells/organisms potentiating toxic effects. Several reports and reviews on nanoparticle safety state that there are knowledge gaps concerning the ability of nanoparticles to act as vectors of chemicals, micro-organisms and interactions with other stressors [22, 25].

Release of (toxic) heavy metal ion species from metal containing nanoparticles under environmental conditions should be taken into account in their ecotoxicity evaluation. Indeed, it has been shown that speciation influences mobility [54], bioavailability and (eco)toxicity of heavy metals in soils [55] as well as in aquatic systems [56-58]. Also, metal solubility may be changed by organisms: initially insoluble forms of heavy metals may become bioavailable due to the direct contact between bacteria and soil particles [55, 59]. There are emerging data on solubility effects of metal oxide nanoparticles to environmentally relevant organisms. For the algae *Pseudokirchneriella subcapitata* the toxicity of nano and bulk ZnO was shown to be attributed solely to dissolved Zn [60]. Analogously, recent results by Heinlaan *et al.* [61] showed that toxicity of CuO and ZnO to bacteria and crustaceans *Thamnocephalus platyurus* was largely caused by bioavailable Cu and Zn ions, although the solubility of CuO and ZnO in water is low (pK_sp_ values are 16.66 and 20.35, respectively). The same was demonstrated for ZnO and CuO (nano)particles for the algae *Pseudokirchneriella subcapitata* by Aruoja *et al.* (submitted to Science of the Total Environment).

## 3. Biotests for Ecotoxicological Evaluation of Metal Oxide Nanoparticles

Nel *et al.* [7] have stressed the importance of pragmatic and mechanism-based approach in testing the potential harmful effects of nanomaterials and three key elements of nanoparticles toxicity screening strategies have been outlined in [62]: i) physicochemical characterization (size, surface area, shape, solubility, aggregation), elucidation of biological effects involving ii) *in vitro* and iii) *in vivo* studies.

As *in vivo* experiments are expensive, slow and ethically questionable there is a strong demand for low-cost high-throughput *in vitro* toxicity assays without reducing the efficiency and reliability of the risk assessment. Moreover, *in vitro* studies allow detailed examination under controlled conditions of various factors involved in toxicity, such as nanoparticles attachment, intracellular localization, changes in gene and protein expression, organelles and membrane structure, viability and cell cycle [63]. In addition, this approach is adhering to 3Rs strategy (replacement, reduction, refinement) introduced by Russel and Burch [64] that means the reduction of the use of laboratory vertebrate animals (mammals, fish) in scientific studies as well as in the legislature-driven research. Indeed, it has been shown that bacterial and invertebrate animal models can be used not only in ecotoxicology [65] but also for toxicological research [67]. In their review article “Assessing the Risks of Manufactured Nanomaterials” Wiesner *et al.* [68] state that “Microbial ecotoxicology is a particularly important consideration in elucidating cytotoxicity mechanisms that could be extrapolated to eukaryotic cells. Moreover, because microorganisms are the foundation of all known ecosystems, serving as the basis of food webs and the primary agents for global biogeochemical cycles, they are important components of soil health. Microorganisms could serve as potential mediators of nanoparticle transformations that affect their mobility and (eco)toxicity”. Crane and Handy [69] have concluded that the general approach for risk assessment of nanoparticles may involve existing regulatory ecotoxicity tests. As no single test or species of living organism show uniform sensitivity to all chemical compounds, the battery of biotests with different sensitivity profiles is often recommended and used to assure adequate evaluation of the ecotoxicological situation. Due to the complexity of ecosystems the ecotoxicological hazard assessment is more informative/predictive if the battery involves organisms of different trophic levels [70, 71]. For example, in regulatory testing of ecotoxicological hazard of pure chemicals, the following test species of different food-web level are recommended: fish (OECD Guideline 203), *Daphnia* (OECD Guidelines 202, 211), algae (OECD Guideline 201). These aquatic species are also often used for monitoring of water quality and hazard assessment of wastewaters since they respond in a predictable manner to the presence of most types of pollutants. For example, *Daphnia* flagged the pollution in the River Meuse that was not detected by direct chemical measurements of water quality [72]. Several criteria have to be taken into account to select ecotoxicological assays: i) the trophic level of the test organism and its sensitivity; ii) the recognition of the test by international standardization organizations; iii) the simplicity of the test; iv) its commercial availability and v) the necessary equipment and the running costs.

**Figure 2.**
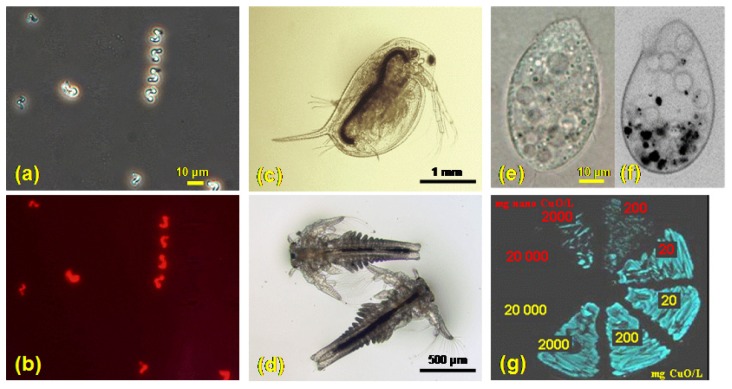
Test organisms used for aquatic risk assessment of chemicals and now increasingly used in nanoecotoxicology: algae *Pseudokirchneriella subcapitata* cells under phase-contrast (a) and fluorescence (b) microscope; nanoCuO accumulation visible in the gut of crustaceans *Daphnia magna* (c) and *Thamnocephalus platyurus* (d) after exposure to nano CuO; protozoan *Tetrahymena thermophila* before (e) and after (f) exposure to nano CuO; naturally luminescent bacteria *Vibrio fischeri* (g) - growth of bacteria on agar medium after pre-incubation for 8 h in the suspensions of nano CuO and bulk CuO. Photo is taken in dark.

Accordingly, a simplified multitrophic test battery for evaluation of hazard of nanoparticles to aquatic ecosystems could involve algae (primary producers), crustaceans and/or protozoa (consumers) and bacteria (decomposers) (Figure 2) in acute and if possible also in chronic tests:

- The algae *Pseudokirchneriella subcapitata* (formerly *Selenastrum capricornutum*) (Figure 2a,b) is a relevant model organism for predicting the toxic hazard to primary producers and algal growth inhibition assay is widely used in aquatic risk assessment [73]. The assay has been also adapted for turbid samples, for example soil suspensions [74] and could be further developed for nanoparticles. As nanoparticles are shading the light necessary for growth of algae, the appropriate controls should be added to address the shading [28].
- The small aquatic crustacean *Daphnia magna* is considered a “keystone” species in aquatic toxicology for acute and chronic toxicity studies. *Daphnia* has been considered an obvious first choice for test organisms when performing ecotoxicological tests on nanomaterials [24]. The genome of another crustacean *Daphnia pulex* is almost sequenced and there is some toxicogenomic data also for *D. magna* available [75]. As particle-ingesting organisms (Figure 2c) they are very appropriate to test nanoparticles.
- Also another small crustacean, *Thamnocephalus platyurus* (Figure 2d), may be used in screening studies instead of *Daphnia* as both are crustaceans and of comparable sensitivity [61, 73].
- Being ecologically widely spread and particle-ingesting organisms, protozoa are very relevant for nanotoxicology (Figure 2 e, f), particularly the well studied *Tetrahymena pyriformis* and *Tetrahymena thermophila* [71]. TETRATOX database for *T. pyriformis* [76] involves toxicity data for more than 2000 industrial organic compounds. *T. thermophila* could be important for toxicogenomic studies as its macronuclear genome was sequenced in 2006 [77]. Protozoa *T. pyriformis* have been studied for the effects of carbon nanotubes [78] and fullerenols [79]. It has been shown that *T. thermophila* ingested SWNT (single wall nanotubes) and bacteria with no apparent discrimination but at 3.6 mg SWNT/L exposure level the bacterivory became inhibited. Thus, SWNT may move up the food chain and that carbon nanotubes may potentially disrupt the role of ciliates in regulating bacterial populations [80]. Up to now there is no published peer-reviewed data on toxicity of metal oxide nanoparticles to protozoa.
- The former four tests may be done independently of the “culturing/maintenance” burden of live stocks of test species by using commercially available as “Toxkit microbiotests” (MicroBioTests Inc., Nazareth, Belgium).
- Despite of its marine origin, the most widely used bacterium for ecotoxicological studies is naturally luminescent *Vibrio fischeri* (Figure 2g). This bacterial luminescence inhibition assay is rapid, cheap and easy to perform and with a lot of toxicity data available for pure chemicals. Several different luminescence inhibition tests of *V. fischeri* have been developed so far – most of them are designed for analysis of aqueous samples (Microtox^®^ BioTox™, LUMIStox™, ToxAlert™), while only one of the test protocols (Flash Assay) has been successfully used for analysis of suspensions, turbid and colored samples: in this kinetic assay each sample acts as its own reference [81-84]. Flash Assay using *Vibrio fischeri* was recently shown to be a very powerful tool for screening of the toxicity of both, metal oxide as well as organic nanoparticles even in the case of turbidity due to insolubility and/or aggregation of particles [61, 85]. *V. fischeri* Flash Assay has also been miniaturized (96-well microplates) for high throughput testing of nanoparticles [85].

In addition to biotests concerning a single organism species, data on the behavior and effects of nanoparticles in the environmental food-chain would be of primary importance for understanding their overall potential hazard for ecosystems. However, the harmful effects of manufactured nanoparticles on aquatic and soil organisms are largely unknown and the available information concerns mainly fullerenes [86]. For example, using C60 fullerenes as a model, the first report on the impact of synthetic nanoparticles fullerenes on microorganisms in soil was provided in 2007 [87]. Concerning metal oxide nanoparticles, ecotoxicity of TiO_2_ at different aquatic food-chain level is most well documented (Table 1). In general, nanoparticles may sorb on external surfaces of bacteria [88] and probably also on phytoplankton [89] that is food for crustaceans. TiO_2_ particles have been shown to adsorb onto the algal cell surface, resulting in a 2.3-fold increase of cellular weight as referred in [23]. Also, adhesion of nanoparticle aggregates to the exoskeleton of the test organisms is frequently described for the crustacean studies as summarized in [24].

Recently, dietary accumulation, elimination and ecotoxicity of fluorescent quantum dots (carboxylated and biotinylated CdSe/ZnS QDs) were investigated using two aquatic organisms - *Tetrahymena pyriformis*, a single-celled ciliate protozoan, and the rotifer *Brachionus calyciflorus* that preys on it [50]. Although the transfer of QDs was observed, limited bioconcentration and lack of biomagnification may hamper the detection of nanomaterials in invertebrate species. In addition, the used QDs concentrations are unlikely to be encountered in natural environment and natural organic matter may substantially alter behavior and bioavailability of nanoparticles.

## 4. Aggregation of Nanoparticles

Various environmental parameters may lead to aggregation of nanoparticles released in the environment. Nanoparticles tend to aggregate in seawater [86] and in freshwater systems [60]. As nanoparticles tend to aggregate and settle in aqueous suspensions as well as tend to sorb nutrients and to interfere with chemicals/parameters used for evaluation of (eco)toxicity, careful design of sample preparation and choosing of test endpoints as well as including suitable positive controls may be necessary. As some problems are similar to the testing of the soil or sediment suspensions, some lessons may be learned from following studies [74, 81].

The surface properties of nanoparticles are one of the most important factors that govern their stability and mobility as colloidal suspensions or their aggregation into larger particles and deposition in aquatic systems [23]. For TiO_2_, it has been shown that nanoparticle aggregation behavior strongly depended on pH and ionic strength. Also, cationic and anionic species or the presence of humic acids affected the stability of TiO_2_ suspensions [90]. It has thus been suggested [91] that aggregation may have implications on toxicity, which may result in very dissimilar biological activity. It is difficult to evaluate the (eco)toxic impact of aggregation without assessment of the specific surface area which is involved in solubilisation, adsorption and catalytic properties. In addition, in the case of metals, it is well known that reactive chemical metal species depend not only on solubility, but also on the whole set of associated ions and even on slight changes of pH. This might explain discrepancies found in the emerging literature on the aquatic toxicity of metal oxide nanoparticles summarized below.

## 5. Bioavailability of Metals from Metal Oxide Nanoparticles

Bioavailable fractions of metals from metal containing nanoparticles may be studied in a combined approach involving chemical analysis and recombinant metal-specific microbial sensors. Those genetically modified microbial biosensor strains will only produce a response if the toxic compound (for example, heavy metal) crosses the cell biological envelopes and enters the cytoplasmic space, that is if the toxicant is accessible or bioavailable to the sensing system. Mostly those sensors are based on bacteria [92]. In a metal-specific microbial sensor the expression of a reporter gene is controlled by a genetic regulatory unit (receptor), which responds to the given heavy metal, i.e. receptor–reporter concept [93] is used. Most of the regulatory units used in the construction of metal-specific sensor bacteria originate from bacteria that possess natural precisely regulated resistance systems towards heavy metals. As those recombinant bacterial cells are specially modified to respond to intracellular subtoxic concentrations of heavy metals by increasing an easily detectable signal, for example luminescence, they are very promising tools to detect bioavailable heavy metals. A number of recombinant bacterial sensors for Cd, As, Sb, Cr, Cu, Hg, Zn and Pb reviewed in [94, 95] have been developed.

Metal-specific recombinant bacterial sensors have been constructed and used for the determination of bioavailable fractions of inorganic mercury [96, 97], organomercurials [98], zinc, cadmium, cobalt and lead [99], cadmium and lead [97, 100], nickel [101] and chromate [97]. This approach based on biovailability of heavy metals in different environmental matrices has been successfully used for environmental hazard evaluation in soils [55, 59, 102]. As dissolved metal concentrations may be very low, sensitized bacterial heavy metal sensors based on knock-out mutants of *Pseudomonas putida* metal transporters have been constructed and their increased sensitivity towards heavy metals demonstrated [103]. Recently, recombinant sensor bacteria have been used in totally novel context: for the evaluation of the bioavailability of zinc and copper in aqueous suspensions of ZnO and CuO nanoparticles and comparing that with ecotoxic values of respective metal ions to crustaceans [61] and microalgae (Aruoja *et al.*, submitted to Science of the Total Environment).

## 6. Ecotoxicity of TiO_2_, ZnO and CuO Nanoparticles: Emerging Data

### 6.1. TiO_2_

In the case of bacteria, high concentrations of nano TiO_2_ (66 nm advertised particle size) were needed to inhibit the growth of *Escherichia coli*: 5,000 mg TiO_2_/L was reducing the growth of bacteria by 72% whereas *Bacillus subtilis* was slightly more sensitive (1,000 mg TiO_2_ resulted in 75% growth inhibition) [40]. For the marine bacterium *Vibrio fischeri* no toxic effect was observed (30-min EC50 > 20,000 mg TiO_2_/L) [61].

According to Warheit *et al.* [104] the *Daphnia magna* 48h EC_50_ values and rainbow trout (*Oncorhynchus mykiss*) 96 h LC_50_ values for fine TiO_2_ particles (median particle sizes of ∼380 nm) and ultrafine TiO_2_ particles (median particle sizes of ∼140 nm; 90 wt% TiO_2_, 7% alumina, and 1% amorphous silica), based on nominal concentrations were >100 mg/L. The algae 72 h EC_50_ values based on inhibition of growth were 16 mg/L for fine TiO_2_ particles and 21 mg/L for ultrafine TiO_2_ particles [104]. Thus, according to [104] results of the above described aquatic toxicity screening studies demonstrated that ultrafine TiO_2_ exhibited low concern for aquatic hazard using the *Daphnia magna* as well as using the rainbow trout and exhibited medium concern in a 72 h acute test using the green algae *Pseudokirchneriella subcapitata.* Analogously, nano nor bulk TiO_2_ showed no concern for aquatic hazard using crustaceans *D. magna* and *Thamnocephalus platyurus* (LC_50_>10,000 mg TiO_2_/L) (tested without illumination) [61]. In another study, titanium dioxide nanoparticles showed some sublethal toxic effects (including oxidative stress) in rainbow trout when exposed to low levels (0.1-1.0 mg TiO_2_/L) during up to 14 days [105]. When filtering TiO_2_ nanoparticles suspension (0.22 μm) to avoid the interference of aggregates, Lovern and Klaper [106] reported high acute toxicity to *D. magna* (LC_50_=5.5 mg TiO_2_/L). However, when nanoparticles of TiO_2_ were illuminated before testing, acute toxic effects for daphnids occurred at lower level (1.5-3 mg TiO_2_/L) [28]. Indeed, in addition to target organism and exposure time, two major abiotic parameters seem to be involved in the actual (eco)toxicity of TiO_2_: particle size/aggregation and illumination.

### 6.2. ZnO

Ecotoxicity of nano zinc oxide has been shown to be quite comparable to bulk ZnO. For *Vibrio fischeri* the 30-min EC_50_ was ∼ 2 mg ZnO/L, for *D. magna* the 48-h LC_50_ was in the range of ∼3-9 mg ZnO/L, for *T. platyurus* 24-h LC_50_ was ∼0.2 mg ZnO/L [61] and for freshwater algae *Pseudokirchneriella subcapitata* 72-h EC_50_ value was ∼0.08 mg ZnO/L [60]. As discussed also above, soluble Zn^2+^ from ZnO seems to be the main factor of its (eco)toxicity as proven by several studies. Franklin *et al.* [60] studied the toxicity of ZnO nanoparticles to algae *P. subcapitata* while also determining the concentration of dissolved Zn ions derived from ZnO. Using a physical method, i.e. dialysis membrane with a pore size of about 1 nm (permeable to Zn ions but not to ZnO particles) they showed that both nano and bulk ZnO suspensions yielded similar dissolved Zn concentrations. Analogously, using Zn-sensor bacteria and comparing the ecotoxicity of Zn^2+^ and ZnO and nano ZnO, Heinlaan *et al.* [61] showed that toxicity of (nano) ZnO to crustaceans *Daphnia magna* and *Thamnocephalus platyurus* and bacteria *Vibrio fischeri* was attributed to soluble Zn^2+^. For the algae *P. subcapitata*, identical results were obtained by Aruoja *et al.* (submitted to Science of the Total Environment).

### 6.3. CuO

Copper salts have been used as biocides for a long time and the short-term toxic effect of copper is used for antifouling in marine paints, free copper ions released preventing attachment of organisms to the vessel [107]. Differently from zinc oxide, nano copper oxide has been shown to be remarkably more toxic than bulk CuO: bulk CuO was toxic to *V. fischeri* at ∼ 4,000 mg /L, nano CuO at ∼ 80 mg/L. To *D. magna* and *T. platyurus* the toxicity of bulk CuO was ∼100-150 mg/L and nano CuO ∼2-3 mg/L. Analogously to ZnO, the toxicity of CuO to *V. fischeri* and *T. platyurus* [61] as well as to algae *Pseudokirchneriella subcapitata* (Aruoja *et al.*, submitted to Science of the Total Environment) was largely explained by soluble Cu^2+^ as proved by the recombinant Cu-sensor bacteria.

## 7. Conclusions

Despite the importance of metal oxide nanoparticles, the data on their ecotoxicity are rare (Table 1). The heterogeneity of emerging ecotoxicity data for metal oxide nanoparticles shows the need for additional studies but also for standardization/modification of the respective test protocols. The toxicity mechanisms of metal oxide nanoparticles may be related to either soluble ions or particle properties or both. In all cases, aggregation and chemical speciation play a leading role in their (eco)toxicity. Using recombinant metal-specific bacterial biosensors and a suite of multitrophic invertebrate, algal and bacterial toxicity assays in tandem, new scientific knowledge has been and can be obtained in the future on the respective role of ionic species and of particles. Currently, advances of biosensor molecular biology have led to the development of new recombinant microorganisms for research on bioavailability and (eco)toxicity of heavy metals as well as other mechanisms of toxicity. Their use in nanoecotoxicology is under development.

## Figures and Tables

**Table 1.** Number of peer-reviewed papers for selected metal oxides found in ISI Web of Science for years 1980-2008. Combinations of key-words comprising “nano*”, “toxic*” and “ecotoxic*” inserted for the search in “topic” were used as indicated in Table 1. Search was performed on 4.07.2008.

**Metal oxide**	**Number of papers**			**Organisms**

	+nano*	+nano*	+nano*	
		+toxic*	+ecotoxic*	

TiO_2_	12390	114	6	Bacteria, fungi, crustaceans, microalgae, fish, plants
ZnO	6314	21	3	Bacteria, crustaceans
CuO	914	4	1	Bacteria, crustaceans
Al_2_O_3_	5504	14	1	Fish embryos
SiO_2_	10027	29	1	Text concerns occupational health and safety problems
Fe_2_O_3_	2627	20	0	
ZrO_2_	2599	9	0	

## References

[1] Directive 2006/121/EC of the European Parliament and of the Council of 18 December 2006amending Council Directive 67/548/EEC on the approximation of laws, regulations and administrative provisions relating to the classification, packaging and labelling of dangerous substances in order to adapt it to Regulation (EC) No 1907/2006 concerning the Registration, Evaluation, Authorisation and Restriction of Chemicals (REACH) and establishing a European Chemicals Agency..

[2] Franco A., Hansen S.F., Olsen S.I., Butti L. (2007). Limits and prospects of the “incremental approach” and the European legislation on the management of risks related to nanomaterials. Regul. Toxicol. Pharm..

[3] Ryan J.N., Elimelech M. (1996). Colloid mobilization and transport in groundwater. Colloids Surf., A..

[4] Lead J.R., Wilkinson K.J. (2006). Natural aquatic colloids: current knowledge and future trends. Environ. Chem..

[5] Englert N. (2004). Fine particles and human health—a review of epidemiological studies. Toxicol. Lett..

[6] Wigginton N.S., Haus K.L., Hochella M.F. (2007). Aquatic environmental nanoparticles. Critical Review. J. Environ. Monit..

[7] Nel A., Xia T., Mädler L., Li N. (2006). Toxic potential of materials at nanolevel. Science.

[8] Maynard A., Michelson E. (2006). The Nanotechnology Consumer Products Inventory..

[9] Tratnyek P.G., Johnson R.L. (2006). Nanotechnologies for environmental cleanup. Nano Today.

[10] Zhang W. (2003). Nanoscale iron particles for environmental remediation: An overview. J. Nanoparticle Res..

[11] Serpone N., Dondi D., Albini A. (2007). Inorganic and organic UV filters: Their role and efficacy in sunscreens and suncare products. Inorg. Chim. Acta..

[12] Yuranova T., Laub D., Kiwi J. (2007). Synthesis, activity and characterization of textiles showing self-cleaning activity under daylight irradiation. Catal. Today.

[13] Reijnders L. (2006). Cleaner nanotechnology and hazard reduction of manufactured nanoparticles. J. Cleaner Prod..

[14] Cai R., Van G.M., Aw P.K., Itoh K. (2006). Solar-driven self-cleaning coating for a painted surface. C.R. Chim..

[15] Blake D.M., Maness P-C., Huang Z., Wolfrum E.J., Jacoby W.A., Huang J. (1999). Application of the photocatalytic chemistry of titanium dioxide to disinfection and the killing of cancer cells. Sep. Purif. Methods..

[16] Zhou K., Wang R., Xu B., Li Y. (2006). Synthesis, characterization and catalytic properties of CuO nanocrystals with various shapes. Nanotechnology.

[17] Chang H., Jwo C.S., Lo C.H., Tsung T.T., Kao M.J., Lin H.M. (2005). Rheology of CuO nanoparticle suspension prepared by ASNSS. Rev. Adv. Mater.Sci..

[18] Hardman R. (2006). A toxicologic review of quantum dots: toxicity depends on physicochemical and environmental factors. Environ. Health Perspect..

[19] Oberdörster E. (2004). Manufactured nanomaterials (fullerens, C60) induce oxidative stress in the brain of juvenile largemouth bass. Environ. Health Perspect..

[20] Lockman P., Oyewumi M., Koziara J., Roder K.E., Mumper R.J., Allen D.D. (2003). Brain uptake of thiamine-coated nanoparticles. J. Control. Rel..

[21] Oberdörster G., Oberdörster E., Oberdörster J. (2005). Nanotoxicology: an emerging discipline evolving from studies of ultrafine particles. Environ. Health Perspect..

[22] (2004). Nanoscience and nanotechnologies: opportunities and uncertainties. http://www.nanotec.org.uk/finalReport.htm.

[23] Navarro E., Baun A., Behra R., Hartmann N.B., Filser J., Miao A.-J., Quigg A., Santschi P.H., Sigg L. (2008). Environmental behavior and ecotoxicity of engineered nanoparticles to algae, plants, and fungi. Ecotoxicology.

[24] Baun A., Hartmann N.B., Grieger K., Kusk K.O. (2008). Ecotoxicity of engineered nanoparticles to aquatic invertebrates: a brief review and recommendations for future toxicity testing. Ecotoxicology.

[25] Moore M.N. (2006). Do nanoparticles present ecotoxicological risks for the health of the aquatic environment?. Environ. Int..

[26] Oberdörster E., Zhu S., Blickley M., McClellan-Green P., Haasch M.L. (2006). Ecotoxicology of carbon-based engineered nanoparticles: Effects of fullerene (C60) on aquatic organisms. Carbon.

[27] Donaldson K., Tran C.L. (2002). Inflammation caused by particles and fibers. Inhal. Toxicol..

[28] Hund-Rinke K., Simon M. (2006). Ecotoxic effect of photocatalytic active nanoparticles (TiO_2_) on algae and daphnids. Environ. Sci. Pollut. Res..

[29] Kelly S.A., Havrilla C.M., Brady T.C., Abramo K.H., Levin E.D. (1998). Oxidative stress in toxicology: established mammalian and emerging piscine model systems. Environ. Health Perspect..

[30] Nel A.E., Diaz-Sanchez D., Li N. (2001). The role of particulate pollutants in pulmonary inflammation and asthma: Evidence for the involvement of organic chemicals and oxidative stress. Curr. Opin. Pulmon. Med..

[31] Singh S., Shi T., Duffin R., Albrecht C., van Berlo D., Höhr D., Fubini B., Martra G., Fenoglio I., Borm P.J.A., Schins R.P.F. (2007). Endocytosis, oxidative stress and IL-8 expression in human lung epithelial cells upon treatment with fine and ultrafine TiO_2_: Role of the specific surface area and surface methylation of the particles. Toxicol. Appl. Pharm..

[32] Rikans L.E., Hornbrook K.R. (1997). Lipid peroxidation, antioxidant protection and aging. Biochim. Biophys. Acta.

[33] Li N., Sioutas C., Cho A., Schmitz D., Misra C., Sempf J., Wang M., Oberley T., Froines J., Nel A. (2003). Ultrafine particulate pollutants induce oxidative stress and mitochondrial damage. Environ. Health Perspect..

[34] Kastan M. (2007). Our cells get stressed too! Implications for human disease. Blood Cells Mol. Dis..

[35] Unlu E.S., Koc A. (2007). Effects of deleting mitochondrial antioxidant genes on life span. Ann. N. Y. Acad. Sci..

[36] Shvedova A.A., Kisin E.R., Mercer R., Murray A.R., Johnson V.J., Potapovich A.I., Tyurina Y.Y., Gorelik O., Arepalli S., Schwegler-Berry D., Hubbs A.F., Antonini J., Evans D.E., Ku B.K., Ramsey D., Maynard A., Kagan V.E., Castranova V., Baron P. (2005). Unusual inflammatory and fibrogenic pulmonary responses to single walled carbon nanotubes in mice. Am. J. Physiol. Lung Cell. Mol. Physiol..

[37] Brown D.M., Donaldson K., Borm P.J., Schins R.P., Dehnhardt M., Gilmour P., Jimenez L.A., Stone V. (2004). Calcium and ROS-mediated activation of transcription factors and TNF-alpha cytokine gene expression in macrophages exposed to ultrafine particles. Am. J. Physiol. Lung. Cell Mol. Physiol..

[38] Long T.C., Saleh N., Tilton R.D., Lowry G.V., Veronesi B. (2006). Titanium dioxide (P25) produces reactive oxygen species in immortalized brain microglia (BV2): implications for nanoparticles neurotoxicity. Environ. Sci. Technol..

[39] Limbach L.K., Wick P., Manser P., Grass R.N., Bruinink A., Stark W.J. (2007). Exposure of engineered nanoparticles to human lung epithelial cells: influence of chemical composition and catalytic activity on oxidative stress. Environ. Sci. Technol..

[40] Adams L.K., Lyon D.Y., Alvarez P.J.J. (2006). Comparative eco-toxicity of nanoscale TiO_2_, SiO_2_, and ZnO water suspensions. Water Res..

[41] Kim S.C., Lee D.K. (2005). Preparation of TiO_2_-coated hollow glass beads and their application to the control of algal growth in eutrophic water. Microchem J..

[42] Hong J., Ma H., Otaki M. (2005). Controlling algal growth in photo-dependent decolorant sludge by photocatalysis. J. Biosci. Bioeng..

[43] Reeves F.J., Davies S. J., Dodd N. J. F., Jha A. N. (2008). Hydroxyl radicals (•OH) are associated with titanium dioxide (TiO_2_) nanoparticle-induced cytotoxicity and oxidative DNA damage in fish cells. Mutat. Res..

[44] Zhang L., Jiang Y., Ding Y., Povey M., York D. (2007). Investigation into the antibacterial behaviour of suspensions of ZnO nanoparticles (ZnO nanofluids). J. Nanopart. Res..

[45] Pinto E., Sigaud-Kutner T.C.S., Leitaõ M.A.S., Okamoto O.K., Morse D., Colepicolo P. (2003). Heavy metal-induced oxidative stress in algae. J. Phycol..

[46] Brunner T.J., Wick P., Manser P., Spohn P., Grass R.N., Limbach L.K., A. Bruinink A., Stark W.J. (2006). *In vitro* cytotoxicity of oxide nanoparticles: comparison to asbestos, silica, and the effect of particle solubility. Environ. Sci. Technol..

[47] Derfus A.M., Chan W.C., Bhatia S.N. (2004). Probing the cytotoxicity of semiconductor quantum dots. Nano Lett..

[48] SCENIHR (EU Scientific committee on emerging and newly identified health risks) Report 2007..

[49] Kloepfer J.A., Mielke R.E., Nadeau J.L. (2005). Uptake of CdSe and CdSe/ZnS Quantum dots into Bacteria via purine-dependent mechanisms. Appl. Environ. Microbiol..

[50] Holbrook R.D., Murphy K.E., Morrow J.B., Cole K.D. (2008). Trophic transfer of nanoparticles in a simplified invertebrate food chain. Nat. Nanotechnol..

[51] Cheng X., Kan A., Tomson M. (2004). Napthalene adsorption and desorption from aqueous C60 fullerene. J. Chem. Eng. Data.

[52] Xia T., Korge P., Weiss J.N., Li N., Venkatesen M.I., Sioutas C., Nel A. (2004). Quinones and aromatic chemical compounds in particulate matter induce mitochondrial dysfunction: Implications for ultrafine particle toxicity. Environ. Health Perspect..

[53] Baun A., Sørensen S.N., Rasmussen R.F., Hartmann N.B., Koch C.B. (2008). Toxicity and bioaccumulation of xenobiotic organic compounds in the presence of aqueous suspensions of aggregates of nano-C60. Aquat. Toxicol..

[54] François M., Dubourguier H.C., Li D., Douay F. (2004). Prediction of heavy metal solubility in agricultural topsoils around two smelters by the physico-chemical parameters of the soils. Aquat. Sci..

[55] Kahru A., Ivask A., Kasemets K., Põllumaa L., Kurvet I., Francois M., Dubourguier H.C. (2005). Biotests and biosensors in ecotoxicological risk assessment of field soils polluted with zinc, lead and cadmium. Environ. Toxicol. Chem..

[56] Kim S.D., Ma H., Allen H.E., Cha D.K. (1999). Influence of dissolved organic matter on the toxicity of copper to *Ceriodaphnia dubia*: Effect of complexation kinetics. Environ. Toxicol. Chem..

[57] Long K. E., Van Genderen E. J., Klaine S. J. (2004). The effects of low hardness and pH on copper toxicity to *Daphnia magna*. Environ. Toxicol. Chem..

[58] Witters H. E. (1998). Chemical Speciation Dynamics and Toxicity Assessment in Aquatic Systems. Ecotoxicol. Environ. Saf..

[59] Ivask A., Francois M., Kahru A., Dubourguier H.C., Virta M., Douay F. (2004). Recombinant luminescent bacterial sensors for the measurement of bioavailability of cadmium and lead in soils polluted by metal smelters. Chemosphere.

[60] Franklin N., Rogers N., Apte S., Batley G., Gadd G., Casey P. (2007). Comparative toxicity of nanoparticulate ZnO, bulk ZnO, and ZnCl2 to a freshwater microalga (*Pseudokirchneriella subcapitata*): the importance of particle solubility. Environ. Sci. Technol..

[61] Heinlaan M., Ivask A., Blinova I., Dubourguier HC., Kahru A. (2008). Toxicity of nanosized and bulk ZnO, CuO and TiO_2_ to bacteria *Vibrio fischeri* and crustaceans *Daphnia magna* and *Thamnocephalus platyurus*. Chemosphere.

[62] Oberdörster G., Maynard A., Donaldson K., Castranova V., Fitzpatrick J., Ausman K., Carter J., Karn B., Kreyling W., Lai D., Olin S., Monteiro-Riviere N., Warheit D., Yang H. (2005). ILSI Research Foundation/Risk Science Institute Nanomaterial Toxicity Screening Working Group. Principles for characterizing the potential human health effects from exposure to nanomaterials: elements of a screening strategy. Part Fibre Toxicol..

[63] Panessa-Warren B.J., Warren J.B., Wong S.S., Misewich J.A. (2006). Biological cellular response to carbon nanoparticles toxicity. J. Phys: Condens. Matter.

[64] Russel W.M.S., Burch R.L. (1959). The principles of Humane Experimental Technique..

[65] Hutchinson T.H., Barrett S., Buzby M., Constable D., Hartmann A., Hayes E., Huggett D., Laenge R., Lillicrap A.D., Straub J.O., Thompson R.S. (2003). A strategy to reduce the numbers of fish used in acute ecotoxicity testing of pharmaceuticals. Environ. Toxicol. Chem..

[66] Kahru A. (2006). Ecotoxicological tests in non-ecotoxicological research: contribution to 3Rs. Use of luminescent photobacteria for evaluating the toxicity of 47 MEIC reference chemicals. ALTEX.

[67] Kahru A., Drews M., Põllumaa L., Kasemets K., Veidebaum T., Kogerman P. (2005). Toxicity of nanoscale cationic polymers *in vitro* and *in vivo*. ALTEX.

[68] Wiesner M.R., Lowry G.V., Alvarez P., Dionysiou D., Biswas P. (2006). Assessing the risks of manufactured nanomaterials. Environ. Sci. Technol..

[69] Crane M., Handy R.D. (2007). An assessment of regulatory testing strategies and methods for characterizing the ecotoxicological hazards of nanomaterials..

[70] Blaise C. (1991). Microbiotests in aquatic ecotoxicology: characteristics, utility, and prospects. Environ. Toxicol. Water Qual..

[71] Blaise C. (1998). Microbiotesting: An expanding field in aquatic toxicology. Ecotoxicol. Environ. Saf..

[72] De Hoogh C.J., Wagenvoort A. J., Jonker F., van Leerdam J. A., Hogenboom A.C. (2006). HPLC-DAD and Q-TOF MS techniques identify cause of *Daphnia* biomonitor alarms in the river Meuse. Environ. Sci. Technol..

[73] Blinova I. (2004). Use of freshwater algae and duckweeds for phytotoxicity testing. Environ. Toxicol..

[74] Aruoja V., Kurvet I., Dubourguier H.C., Kahru A. (2004). Toxicity testing of heavy metal polluted soils with algae *Selenastrum capricornutum*: a soil suspension assay. Environ. Toxicol..

[75] Soetaert A., Moens L.N., Van der Ven K., Van Leemput K., Naudts B., Blust R., De Coen W. M. (2006). Molecular impact of propiconazole on *Daphnia magna* using a reproduction-related cDNA array. Comp. Biochem. Physiol. C: Pharmacol. Toxicol..

[76] Schultz T.W. (1997). TETRATOX: *Tetrahymena pyriformis* population growth impairment endpoint-A surrogate for fish lethality. Toxicol. Methods.

[77] Eisen J.A., Coyne R.S., Wu M., Wu D., Thiagarajan M., Wortman J.R., Badger J.H., Ren Q., Amedeo P., Jones K.M., Tallon L.J., Delcher A. L., Salzberg S.L., Silva J. C., Haas B.J., Majoros W. H., Farzad M., Carlton J.M., Smith R.K., Garg J., Pearlman R.E., Karrer K M., Sun L., Manning G., Elde N.C., Turkewitz A. P., Asai D.J., Wilkes D.E., Wang Y., Cai H., Collins K., Stewart B. A., Lee S.R., Wilamowska K., Weinberg Z., Ruzzo W.L., Wloga D., Gaertig J., Frankel J., Tsao C.-C., Gorovsky M.A., Keeling P.J., Waller R.F., Patron N.J., Cherry J.M., Stover N.A., Krieger C.J., del Toro C., Ryder H.F., Williamson S.C., Barbeau R.A., Hamilton E.P., Orias E. (2006). Macronuclear genome sequence of the ciliate *Tetrahymena thermophila*, a model eukaryote. PLoS Biol.

[78] Zhu Y., Ran T., Li Y., Guo J., Li W. (2006). Dependence of the cytotoxicity of multi-walled carbon nanotubes on the culture medium. Nanotechnology.

[79] Zhao Q.-F., Zhu Y., Ran T.-C., Li J.-G., Li Q.-N., Li W.-X. (2006). Cytotoxicity of fullerenols on *Tetrahymena pyriformis*. Nuclear Sci. Tech..

[80] Ghafari P., St-Denis C.H., Power M.E., Jin X., Tsou V., Mandal H.S., Bols N.C., Tang X. (2008). Impact of carbon nanotubes on the ingestion and digestion of bacteria by ciliated protozoa. Nat. Nanotechnol..

[81] Lappalainen J., Juovinen R., Vaajasaari K., Karp M. (1999). A new flash method for measuring the toxicity of solid and colored samples. Chemosphere.

[82] Põllumaa L., Kahru A., Eisenträger A., Reiman R., Maloveryan A., Rätsep A. (2000). Toxicological investigation of soils with the solid-phase Flash assay: comparison with other ecotoxicological tests. ATLA.

[83] Põllumaa L., Kahru A., Manusadzianas L. (2004). Biotest- and chemistry-based hazard assessment of soils, sediments and solid wastes. J. Soils Sediments..

[84] Heinlaan M., Kahru A., Kasemets K., Kurvet I., Waterlot C., Sepp K., Dubourguier H.-C., Douay F. (2007). Rapid screening for soil ecotoxicity with a battery of luminescent bacteria tests. ATLA.

[85] Mortimer M., Kasemets K., Kurvet I., Heinlaan M., Kahru A. (2008). Kinetic *Vibrio fischeri* bioluminescence inhibition assay for study of toxic effects of nanoparticles and colored/turbid samples. Toxicol. in Vitro.

[86] Handy R.D., Kammer F.V.D., Lead J.R., Hassellöv M., Owen R., Crane M. (2008). The ecotoxicity and chemistry of manufactured nanoparticles. Ecotoxicology.

[87] Tong Z., Bischoff M., Nies L., Applegate B., Turco R.F. (2007). Impact of fullerene (C60) on a soil microbial community. Environ. Sci. Technol..

[88] Li B., Logan B.E. (2004). Bacterial adhesion to glass and metal-oxide surfaces. Colloids Surf. B.

[89] Rhee G.Y., Thomson P.A. (2004). Sorption of hydrophobic organic contaminants and trace metals on phytoplankton and implications for toxicity assessment. J. Aqua. Ecosys. Health.

[90] Ottofuelling S., Kammer F.v.d., Hofmann T. (2007). Nanoparticles in the aquatic environment – aggregation behavior of TiO2 nanoparticles studied in a simplified aqueous test matrix (SAM). Geophys. Res. Abs..

[91] Baveye P., Laba M. (2008). (2008) Aggregation and toxicology of titanium dioxide nanoparticles Environ. Health Perspect..

[92] Jansson J K. (2003). Marker and reporter genes: illuminating tools for environmental microbiologists – Curr. Opin. Microbiol..

[93] Lewis J.C., Feltus A., Ensor C.M., Ramanathan S., Daunert S. (1998). Applications of reporter genes. Anal. Chem..

[94] Köhler S., Belkin S., Schmid R.D. (2000). Reporter gene bioassays in environmental analysis. Fresenius J. Anal. Chem..

[95] Daunert S., Barrett G., Feliciano J.S., Shetty R., Shrestha S., Smith-Spencer W. (2000). Genetically engineered whole-cell sensing systems: coupling biological recognition with reporter genes. Chem. Rev..

[96] Selifonova O., Burlage R., Barkay T. (1993). Bioluminescent sensors for detection of bioavailable Hg(II) in the environment. Appl. Environ. Microbiol..

[97] Ivask A., Virta M., Kahru A. (2002). Construction and use of specific luminescent recombinant bacterial sensors for the assessment of bioavailable fraction of cadmium, zinc, mercury and chromium in the soil. Soil Biol. Biochem..

[98] Ivask A., Hakkila K., Virta M. (2001). Detection of organomercurials with sensor bacteria. Anal. Chem..

[99] Corbisier P., Thiry E., Diels L. (1996). Bacterial biosensors for the toxicity assessment of solid wastes. Environ. Toxicol. Water Qual..

[100] Tauriainen S., Karp M., Chang W., Virta M. (1998). Luminescent bacterial sensor for cadmium and lead. Biosens. Bioelectron..

[101] Tibazarwa C., Corbisier P., Mench M., Bossus A., Solda P.v., Mergeay M., Wyns L., van der Lelie D. (2001). A microbial biosensor to predict bioavailable nickel and its transfer to plants. Environ. Pollut..

[102] Bernaus A., Gaona X., Ivask A., Kahru A., Valiente M. (2005). Analysis of sorption and bioavailability of different species of mercury on model soil components using XAS techniques and sensor bacteria. Anal. Bioanal. Chem..

[103] Leedjärv A., Ivask A., Kahru A., Virta M., Xiang H., Akieh N.G., Vuorio A.-M., Jokinen T., Sillanpää M. (2007). Improvement of bacterial bioreporters to detect zinc, cadmium and lead in environmental samples.

[104] Warheit D.B., Hoke R.A., Finlay C., Donner E.M., Reed K.L., Sayes C.M. (2007). Development of a base set of toxicity tests using ultrafine TiO2 particles as a component of nanoparticle risk management. Toxicol. Lett..

[105] Federici G., Shaw B.J., Handy R.D. (2007). Toxicity of titanium dioxide nanoparticles to rainbow trout (Oncorhynchus mykiss): Gill injury, oxidative stress, and other physiological effects. Aquat. Toxicol..

[106] Lovern S., Klaper R. (2006). *Daphnia magna* mortality when exposed to titanium dioxide and fullerene nanoparticles. Environ. Toxicol. Chem..

[107] Tubbing D.M.J., Admiraal W., Cleven R.F.M.J., Iqbal M., Van de Meent D., Verweij W. (1994). The contribution of complexed copper to the metabolic inhibition of algae and bacteria in synthetic media and river water. Water Res..

